# Attention placebo control in randomized controlled trials of psychosocial interventions: theory and practice

**DOI:** 10.1186/s13063-015-0679-0

**Published:** 2015-04-11

**Authors:** Lukka Popp, Silvia Schneider

**Affiliations:** Department of Clinical Child and Adolescent Psychology, Faculty of Psychology, Ruhr-Universität Bochum, Bochum, Germany

**Keywords:** Attention placebo control, Randomized controlled trial, Ethics, Psychosocial intervention

## Abstract

Attention placebo control (APC) is considered a highly valid control condition when conducting trials of social interventions. Unfortunately, an appropriate APC condition is rarely used. This letter discusses the tension between methodological and ethical requirements of an APC group in psychosocial interventions based on our experiences with a randomized controlled efficacy study of a parent training program. To prevent negative side effects and high drop-out rates, feasible and accepted attention control conditions are discussed. The paradigms of placebo research must be adapted to the special challenges of psychosocial intervention research.

**Trial registration:** Clinicaltrials.gov NCT02313493: registered 10 December 2014

## Findings

Several researchers claim that psychosocial intervention research should not only rely on the classical design of active treatment versus waitlist control group, but should also include an attention placebo control (APC) arm to test the specific effect of the psychosocial treatment [1]. However, compared to drug treatment research, there is no ‘gold standard’ for a feasible APC condition that would meet the methodological requirements of psychosocial intervention research. This letter discusses the difficulties we encountered in our research on prevention-oriented parent training. In particular, we examine the problems of conducting an APC; that is an intervention that mimics the theoretically inactive elements, but not the active elements of a parent training program. For further discussion on the identification of empirically supported treatments see [2].

Researchers rarely use APCs in the field of parent training. A review of the effectiveness of group-based parent education programs on behavioral problems of 3- to 10-year-old children found that out of 255 such studies none included an APC condition [3]. The same is true for parenting trainings with younger children [4]. To our knowledge, there is no methodological standard for APCs in psychosocial trials. In the following, we discuss different ideas of how to design APCs that meet the ethical and methodological demands of psychosocial intervention research.

In the study described here, the experimental condition was a parent training to improve parenting skills and the psychological well-being of expecting first-time parents and their babies. A discussion group moderated by a psychologist was chosen as the APC condition. Here, the participants were encouraged to share information and exchange experiences and worries about becoming a parent. The intervention group and the APC discussion group were equivalent concerning the number of contacts, time of start and duration, peer support and presence of a professional, but not regarding the content of the specific parent training under study. When we developed the study, four studies were already running in UK and Australia comparing prevention-oriented parent training with care as usual or waitlist. To deepen the understanding of the effectiveness of the parent training program, we wanted to learn how the so-called non-specific (placebo) factors like social support/social attention affect the success of the program. At this point, we were not aware, and more importantly, did not expect that a pure APC arm would result in the problems described here. We questioned whether both the APC and the parenting training group would improve the well-being of becoming first-time parents and therefore would be comparable in regard to clinical equipoise. As far as the additional benefit of one group over the other is not evident, clinical equipoise can be assumed in randomized controlled trails [5]. Professionally-led focus groups that provide social support and attention are shown to be feasible and effective across different mental health difficulties in clinical and community samples [6-9]. We aimed to examine the possible specific factors of the parenting training in addition to the general clinical value of attention and social support.

The study was approved by the Ethics Committee of the Faculty of Psychology at the Ruhr-Universität Bochum on 4 September 2012 (*Votum* 036). Prior to the training, we informed the participants about the purpose of the study and the voluntary nature of their participation. They gave written informed consent before participating. After randomization, 19 couples were allocated to either the parenting training (11 couples) or the APC (8 couples) group. Disproportional drop-out numbers after randomization posed the first methodological problem, which eventually led us to discard the APC group. Specifically, 4 couples (50%) in the APC group and 2 couples (18.2%) allocated to the parent training group dropped out after randomization. Also, the cited reasons for dropping out differed between the two groups. The couples within the APC group dropped out without a reason or because they wanted to participate in the parent training and not in the discussion group. The parents in the training group dropped out because of scheduling difficulties.

Despite the high drop-out rate, four couples remained in the APC group. The dissatisfaction with the content of the discussion group posed an additional reason for our final decision not to continue with this control condition. In the first meeting, the participants questioned the benefits of taking part in the study and asked for active guidance. However, the design of the discussion group condition (APC) called for simply asking the parents what they would be interested in and then encouraging the participants to discuss among themselves. Yet, the participants wanted the psychologists to tell them what they should deal with.

Furthermore, ethical reasons prompted us not to continue the study as originally planned. In particular, we were confronted with parents’ comments and questions that showed risky misunderstandings of childcare, which could have exposed a child to dangerous parenting behavior. For example, some parents had misleading ideas about settling strategies like settling a baby on top of a shaking washing machine. One mother had lost a baby due to sudden infant death (SID) and asked for confirmation regarding advice given in a brochure. We felt ethically obligated to correct false understandings of childcare and to reassure the parents that they had received correct information on SID. We felt that we would be responsible in case parents would leave the group with false and dangerous beliefs discussed within the group but not corrected by a psychologist. Moreover, it was difficult to differentiate between mistaken ideas about parenting that would still be acceptable versus ideas that needed to be commented on and dealt with.

Our experiences demonstrated the contradiction between the ethical and methodological requirements of a placebo control group for psychosocial training interventions like our parenting program. Although we expected that parents would benefit from the social support of other parents and from the monitoring function of the psychologist, it became evident that an undirected discussion group as a placebo control group is neither a feasible nor an ethical alternative to the parenting training for expecting first-time parents. We detected different causes for this outcome.

First, parents were confronted with the randomization of the two intervention arms which in our study design were considered equally attractive. Researchers are ethically required to inform participants about study design and to describe the intervention arms under study. When parents were asked about their motivation to participate in the study, the majority explained that they felt insecure in their future role as parents. Thus, contrary to prior expectations, the APC design did not meet the participants’ expectations of receiving information about parenting. Therefore, the discussion group without active guidance was not as attractive as the parent training group. Second, the APC did not provide a convincing therapeutic rational compared to the experimental group, even though we introduced the APC as a promising intervention with its social support and attention aspects. Furthermore, the disproportionally high drop-out rate within the control condition demonstrates a methodological problem in the group selection. Third, an APC within a parenting program poses an ethical problem: parents might exchange false and harmful information in the discussion group, which the psychologist leading the group must amend.

What are the alternatives? A more appropriate APC should meet the expectations of the target group and must provide a convincing therapeutic rational. For the participants in the present case study, the therapeutic rational would be: (1) social support of other participants and (2) collectively working on solutions for difficulties that can emerge during the transition to parenthood. An agenda based on the participants’ expectations, fixed time frames per topic and communication rules would provide a more clear and conclusive structure for group discussions. To control for attractiveness of APC versus experimental treatment, the program’s credibility before and after the first session should be assessed.

To deal with the contradiction between the ethical and methodological requirements of a placebo control group for psychosocial training interventions like parenting programs, we should look back at the beginning of placebo conditions in research, even drug research. Psychosocial research adopted the use of a placebo condition from drug research except for one important detail - blind participation. If participants would not be aware of the different ingredients of the treatments under comparison (as in drug trials), drop-out after randomization would decline. In fact, common recruitment strategies where treatment conditions are revealed are not suitable for blind randomization. However, instead of randomizing individual participants, a randomization of groups of participants might ensure blinding without inducing unequal drop-out rates. In the case of prevention trials (for example, parenting trainings), researchers may randomize whole institutions instead of individuals through existing prevention infrastructure, such as birth preparation classes or gynecological practices. This type of cluster random allocation may be an ethical and methodologically adequate compromise to maintain blindness.

Although scientific advantages of an APC group are obvious, the design of a control group for a prevention-oriented parenting program calls for alternative methods. One solution might be a dismantling strategy where specific elements of a treatment program are studied separately. For example, the parent training program under investigation includes parent communication skills training and baby-calming techniques. A design with three arms (parent communication versus baby-calming versus combination of both) would help us to evaluate how such components impact the success of a parent training program in expecting first-time parents.

In conclusion, intervention researchers should think out of the box: first, they should consider dismantling strategies to offer more attractive treatment conditions. Second, study designs with cluster randomization might help to avoid drop-outs caused by less desirable placebo conditions. This way more researchers would be encouraged to test for placebo effects and shed light on specific treatment effects as well as on the feasibility of treatments in clinical interventions.

In theory, theory and practice are the same. In practice, they are not.

- Albert Einstein

## Abbreviations

APC: Attention placebo control
SID: Sudden infant death
